# Advantages of vitrification preservation in assisted reproduction and potential influences on imprinted genes

**DOI:** 10.1186/s13148-022-01355-y

**Published:** 2022-11-03

**Authors:** Huanhuan Chen, Lei Zhang, Li Meng, Linlin Liang, Cuilian Zhang

**Affiliations:** 1 Reproductive Medicine Center, Henan Provincial People’s Hospital, People’s Hospital of Zhengzhou University, Henan Provincial People’s Hospital of Henan University, Zhengzhou, Henan China; 2 Henan Joint International Research Laboratory of Reproductive Bioengineering, Zhengzhou, Henan Province China

**Keywords:** Assisted reproduction, Vitrification, Epigenetics, Imprinted genes, DNA methylation

## Abstract

Cryopreservation has important application in assisted reproductive technology (ART). The vitrification technique has been widely used in the cryopreservation of oocytes and embryos, as a large number of clinical results and experimental studies have shown that vitrification can achieve a higher cell survival rate and preimplantation development rate and better pregnancy outcomes. Ovarian tissue vitrification is an alternative method to slow freezing that causes comparatively less damage to the original follicular DNA. At present, sperm preservation mainly adopts slow freezing or rapid freezing (LN2 vapor method), although the vitrification method can achieve higher sperm motility after warming. However, due to the use of high-concentration cryoprotectants and ultra-rapid cooling, vitrification may cause strong stress to gametes, embryos and tissue cells, resulting in potentially adverse effects. Imprinted genes are regulated by epigenetic modifications, including DNA methylation, and show single allele expression. Their accurate regulation and correct expression are very important for the placenta, fetal development and offspring health. Considering that genome imprinting is very sensitive to changes in the external environment, we comprehensively summarized the effect of cryopreservation—especially the vitrification method in ART—on imprinted genes. Animal studies have found that the vitrification of oocytes and embryos can have a significant impact on some imprinted genes and DNA methylation, but the few studies in humans have reported almost no influence, which need to be further explored. This review provides useful information for the safety assessment and further optimization of the current cryopreservation techniques in ART.

## Background

Assisted reproductive technology (ART) is an important part of reproductive medicine, which is used to treat clinical infertility and block genetic diseases [1–3]. It comprises ovarian stimulation, gamete collection, in vitro fertilization (IVF), intracytoplasmic sperm injection (ICSI), in vitro embryo culture, cryopreservation, embryo transfer and trophoblast or blastocyst biopsy. Cryopreservation is a conventional technique for the storage of gametes, embryos or tissues in assisted reproduction. Vitrification is a recently developed cryopreservation method and can successfully preserve human embryos and oocytes since 1999 [4, 5]. Vitrification of ovarian tissue is becoming an alternative to slow freezing [6, 7]. Vitrification can achieve a high success rate [8]; enable the establishment and development of an oocyte bank [9, 10]; and provide opportunities for infertile patients, young female cancer patients and other people who want to retain fertility [11]. However, the vitrification process exerts strong physical stress, with severe osmotic pressure and temperature changes. In addition, the high-concentration cryoprotectants used have cytotoxic effects. The possible adverse effects have been investigated by embryologists and reproductive specialists, including the impact of vitrification on epigenetic modifications [12–15]. The epigenetic modification of the genome is very important for regulating gene expression, cell differentiation and function.

An imprinted gene describes one where two alleles from parents are subject to different epigenetic modifications, resulting in the silencing of one parent allele—that is, the state of single allele expression. One hundred and forty-nine imprinted genes are known in mouse, and 128 imprinted genes were found in humans. The presence of imprinted genes explains why both paternal and maternal genomes are required for eutherian and marsupial mammals [16–18]. The single-parent expression of imprinted genes is essential for normal embryonic development, placental differentiation and prenatal and postpartum growth, in addition to normal neurobehavioral processes and metabolism [19–22]. Imprinting abnormalities are associated with human imprinting syndromes such as Beckwith–Wiedemann syndrome (BWS), Prader–Willi syndrome (PWS) and Angelman syndrome (AS), as well as cancers [23, 24]. The establishment and maintenance of genome imprinting depend on normal epigenetic modifications (DNA methylation, post-translational histone modification, chromatin structure and non-coding RNAs) and their interactions. DNA methylation is the most typical epigenetic modification that controls genomic imprinting [25, 26]. There are differentially methylated regions (DMRs) between the two alleles of imprinted genes, that is, a specific gene sequence has DNA methylation modification in one allele, while the other allele is not modified. The methylation difference between alleles is acquired at gamete stage, and most imprinted DMRs are methylated on the maternally inherited allele [27]. The imprinting of mature gametes is maintained by a specific mechanism during fertilization and the whole preimplantation development [28, 29]. However, a recent study showed that DMRs regulating some imprinted genes exist demethylation and rapid imprinting stabilization during preimplantation embryo development of pigs [30]. In mammals, after the primordial germ cells (PGCs) arrive at the embryonic gonad, the imprinting marks are removed and then reestablished in a sex-specific manner during subsequent gamete development [31, 32]. The timeline and acquisition of DNA methylation at male and female differ considerably. Imprints are established prenatally in pro spermatogonia, whereas, in female gametes, imprint establishment occurs after birth in the growing oocyte as in de novo methylation of other regions [33]. The majority of imprinted genes in human are largely conserved with respect to methylation status and allelic expression in mouse although there are some exceptions. The protection of DNA methylation at imprinting control regions (ICRs) is required in both human and mouse, but the epigenetic modifiers implicated in this maintenance have evolved distinct roles between species [34].

Genome imprinting is sensitive to changes in environmental factors [35]. A number of conventional assisted reproductive technologies, such as hormone stimulation, in vitro culture, cryopreservation and embryo transfer, cause abnormal methylation of imprinted genes [36, 37]. Genomic imprinting can reflect the impact of assisted reproductive technology (ART) on epigenetic stability, to a certain extent [38, 39]. Recent researches show that ART may be associated with an increased incidence rate of human imprinting syndromes AS and BWS, and most ART patients with AS also showed imprinting defect [40–42]. Although the children born with ART had a normal clinical phenotype, they may have carried abnormal methylation in the imprinting control region [43]. At present, the relationship between ART and imprinting disorder is still controversial, which may be related to the low detection rate of imprint disorder, different assisted reproduction schemes and the lack of appropriate control [44, 45].

Cryopreservation of gametes, embryos and tissues, especially by the vitrification method, has been widely used in the field of assisted reproduction, but related safety assessments remain to be conducted. They may interfere with the normal establishment and maintenance of genomic imprinting, lead to abnormal expression of imprinted genes, affect the normal development and function of fetus and placenta, cause adverse pregnancy outcomes and may pose a potential threat to the health of postnatal offspring. Therefore, the relationship between cryopreservation techniques and imprinting disorder should be paid more attention and studied further. This review mainly summarizes the application and advantages of vitrification preservation in assisted reproduction, and its influences on imprinted genes according to the current reports.

## Development of vitrification technique

In assisted reproduction, cryopreservation techniques include slow cooling and rapid cooling. The slow freezing method uses programmable freezers to achieve controlled freezing rates. It relies on the balance between the formation rate of ice crystals and the dehydration rate of cells to prevent the formation of ice crystals in cells. The rapid cooling method requires the use of high concentration of cryoprotectants and additives to quickly dehydrate cells, and then, the device containing cells is directly put into liquid nitrogen to obtain an ultrafast cooling rate. Rapid cooling can be divided into two types, one is called vitrification, and the other is called rapid freezing. Vitrification means that the concentration of cryoprotectant is equal to or higher than 40% (v/v) and a supercooled liquid is converted into a glass-like amorphous solid to prevent ice crystal formation. If ice crystals appear in the solution during freezing or thawing, it is called rapid freezing, not vitrification.

Vitrification is developed on the basis of slow freezing. Vitrification has several advantages over traditional freezing methods: (1) It reduces the time consumed in the process of cryopreservation; (2) using a high-concentration cryoprotectant can shorten the exposure time for the cryoprotectant; (3) it minimizes penetration damage; and (4) it eliminates the cost of expensive slow-freezing equipment and its maintenance. In 1985, W. F. Rall and G. M. Fahy vitrified mouse embryos and they believed vitrification was a feasible method that could replace traditional freezing [46]. In 1989, mouse mature oocytes were vitrified to obtain live offspring [47]. In 1998, Mukaida et al. used the vitrification technique to cryopreserve human embryos, which successfully achieved pregnancy [4]. In 1999, L. Kuleshova et al. successfully vitrified human oocytes using open straw as a carrier rod and obtained a normal offspring through the ICSI technique [5]. In recent years, with the rapid development of ART, the vitrification method has become more and more widely used in the cryopreservation of human gametes, embryos and ovarian tissues and has made great progress [48–51].

## Application and advantages of the vitrification technique in assisted reproduction

### Sperm cryopreservation

Sperm cryopreservation is an important part of ART and male fertility preservation. It can avoid repeated biopsy in patients with azoospermia and oligozoospermia [52]. Cancer patients can also use this technique to maintain their fertility before receiving chemotherapy or radiotherapy. Due to the small volume and large number of mature sperm and good tolerance to freezing conditions, the conventional freezing technique can obtain an acceptable survival rate. E. Isachenko and V. Isachenko et al. have repeatedly explored the vitrification of sperm. It was not until the improvement in plastic containers and the use of sucrose as a cryoprotectant in 2008 that an acceptable survival rate after warming was achieved [53]. In recent years, the vitrification method of sperm has been continuously optimized and can now obtain a higher survival rate and reduce the degree of DNA damage compared with slow-freezing [54, 55]. High-concentration permeable cryoprotectants are cytotoxic and cannot be used for sperm vitrification due to the uniqueness of sperm [56]. The nonpermeable protectant sucrose can be used for rapid freezing and vitrification of sperm, and vitrification can achieve higher sperm motility [57, 58]. At present, studies discussing sperm vitrification are focused on the selection of cryoprotectants and optimization of the cooling process [59]. In the future, we can expect that the vitrification technique is applied to sperm cryopreservation effectively and safely.

### Oocyte and embryo cryopreservation

The vitrification technique has been widely used in oocyte and embryo cryopreservation in recent years and can achieve better clinical outcomes compared with slow-freezing [60, 61]. The method of vitrification and warming of human oocytes was still in the theoretical and highly experimental stage at the end of the last century and entered the stage of practice and experimental test after 2000. It has been applied in assisted reproduction as a standard therapy to preserve fertility since 2013 [62]. Because the growing number of children is conceived by oocyte vitrification without birth defects [63, 64], it appears to be the gold standard technique of preserving fertility in young women [65]. People have recognized many clinical uses and practical advantages of oocyte vitrification, including the following: (1) the fertility preservation of women at risk of fertility loss due to chronic diseases and/or treatment (including Turner syndrome treated with gonadal toxin or radiation, autoimmune diseases and cancer); (2) in the process of assisted reproduction—if appropriate sperm is not obtained, the treatment cycle can be carried out flexibly [66]; (3) reducing the management burden during the treatment cycle of oocyte donation; (4) providing possibilities for patients who choose or need to postpone childbearing age [67]; (5) reducing the cost of infertility treatment; and (6) providing options for patients who are concerned about ethical or legal issues of embryo cryopreservation. Embryo vitrification is the most commonly used method of fertility preservation in ART [68]. If extra embryos are obtained and are of good quality, or when the patient is not suitable for fresh embryo transfer (poor uterine environment, chemotherapy, etc.), the remaining embryos can be cryopreserved and directly transferred after thawing in the next cycle, so as to avoid embryo waste and increase the chance of pregnancy. Embryo vitrification also helps to reduce the rate of multiple births and avoid the occurrence of ovarian hyperstimulation syndrome (OHSS).

### Ovarian tissue cryopreservation (OTC)

According to the GLOBOCAN 2020 database, there were 19.3 million new cancer patients in world in 2020. The new rate of breast cancer has exceeded that of lung cancer, with an estimated 2.3 million new cases, becoming the first cancer to do so, and the mortality rate of breast cancer and cervical cancer is high [69]. With continuous improvements in the medical field, the survival rate of cancer patients has significantly improved [70], but radiotherapy and chemotherapy cause serious fertility damage [71, 72]. How to meet the reproductive needs of such patients has become a global concern. Compared with oocyte and embryo cryopreservation, ovarian tissue cryopreservation does not require ovulation induction and can be directly used before cancer treatment without delaying the treatment time. The cryopreservation of ovarian tissue is not to remove the whole ovary, but generally to cut part of ovarian tissue. Ovarian cortical slices are then cryopreserved through slow freezing or vitrification, which is similar to the principle of oocyte or embryo cryopreservation. Currently, slow freezing is the most frequently used method for cryopreserving human ovarian tissue. OTC is the only fertility preservation method for prepubertal girls, women who cannot delay radiotherapy and chemotherapy and patients with hormone-sensitive tumors [73, 74]. After the transplantation of cryopreserved ovarian tissue, most patients can restore endocrine function. However, only one quarter of women can obtain live birth and the fertilization rate for the obtained oocytes is low. Therefore, relevant cryopreservation methods must be further studied and optimized [75].

Compared with slow freezing, ovarian tissue vitrification causes less damage to the original follicular DNA [76] and has simple operation, low cost and high efficiency. At present, there is no standard method and solution formula for vitrification of ovarian tissue; the size of ovarian tissue, the type and concentration of cryoprotectant and equilibrium time are important factors affecting its efficiency [77]. By 2019, more than 130 infants had obtained live birth through transplantation after ovarian tissue cryopreservation [78], of which two were obtained after vitrification [79, 80]. The effect of vitrification was evaluated by observing the ovarian tissue structure, follicular morphology, intracellular ultrastructure (nucleus, mitochondria, lysosome, etc.), DNA fragment rate of primordial follicles and endocrine function. It was found that the vitrification and transplantation of ovarian tissue were feasible and effective. The specific improvement effect requires further research and long-term follow-up [6].

## Influences of the vitrification technique on imprinted genes in animals and human

### Effects of sperm cryopreservation on imprinted genes

It is very important to establish correct methylation markers at imprinted gene sites during spermatogenesis; abnormal methylation will affect spermatogenesis [81–83]. At present, the main techniques applied to sperm cryopreservation are slow freezing and rapid freezing (LN2 vapor method). Both rapid freezing and slow freezing of porcine sperm can lead to changes in the expression of imprinted gene *Igf2* and DNA-methylation-related genes *Dnmt3a* and *Dnmt3b*, and the addition of cryoprotectants can improve them [84]. The slow freezing of porcine sperm affected the structure of the nucleoprotein and its binding with DNA [85]. It has been found that rapid freezing of mouse sperm hinders the normal DNA demethylation process of fertilized embryos, resulting in abnormal methylation level higher than that in the control group [86]. There was no significant difference in DNA methylation in early embryos obtained by ICSI fertilization of fresh mouse sperm and frozen sperm (without cryoprotectant and placed at − 20 °C) [87]. The short-term refrigeration of horse sperm has little effect on DNA methylation [88], but the DNA methylation level increases abnormally after slow freezing and can be used as a potential index for sperm quality evaluation [89]. Therefore, although non-vitrification techniques are widely applied to sperm cryopreservation, they can have adverse impacts on the imprinted gene and DNA methylation, which need to be further clarified. It is necessary to further optimize the sperm vitrification technique as an alternative to traditional freezing methods.

There are few studies on the effect of human sperm freezing on imprinted genes. Slow freezing of human sperm does not affect the DNA methylation pattern of DMRs related to imprinted genes, including maternal imprinted genes *KCNQ1OT1, SNRPN* and *MEST*, and paternal imprinted genes *MEG3* and *H1*9 [90]. Rapid freezing of human sperm had no significant effect on the methylation of DMRs related to imprinted genes *SNURF, SNRPN* and *UBE3A* contained in chromosome 15q11-q13 [91]. In short, no adverse effect of sperm freezing on imprinted genes was found and there are no relevant studies on sperm vitrification.

### Effects of oocyte vitrification on imprinted genes

Although the survival rate of mature oocytes after vitrification is very high [60, 92], there are still some problems, including the depolymerization of microtubules and spindles [93, 94], premature release of cortical particles [95, 96] and polyspermy fertilization [97]. In recent years, some reports have discussed the effects of oocyte vitrification on imprinted genes. It was found that oocyte vitrification can significantly affect the imprinted genes of oocytes and embryos in mouse, bovine and other animals (Table 1). After the vitrification and warming of mouse MII oocytes, the expression of the imprinted gene *Kcnq1ot1* decreased significantly [98]. Our previous study found that after vitrification of bovine MII oocytes, the expression of imprinted genes *Peg10, Kcnq1ot1* and *Xist* in blastocysts obtained by ICSI increased abnormally [99]. We also found that vitrification of mouse MII oocytes affected the expression of some imprinted genes, including maternal imprinted genes *Peg3, Peg10* and *Igf2r* in oocytes; maternal imprinted genes *Peg3* and *Peg10* and paternal imprinted gene *Gtl2* in IVF 2-cell embryos [100]. However, some studies have reported no significant change in the expression of imprinted genes *Igf2r* and *Gtl2* in 2-cell embryos and blastocysts obtained by parthenogenetic activation after vitrification and warming of mouse MII oocytes [101].

**Table 1:** Effect of gamete cryopreservation on imprinted genes

Reference	Species	Materials	Technology of assessment	Targeted imprinted genes	Conclusions
Kläver et al. [90]	Human	Spermatozoa	Bisulfite conversion	*MEG3, H19, KCNQ1OT1, SNRPN, MEST, ALU, LINE1, VASA, MTHFR*	No significant differences
Khosravizadeh et al. [91]	Human	Spermatozoa	Quantitative methylation specific PCR	*SNURF, SNRPN and UBE3A*	No significant differences
Al-Khtib et al. [105]	Human	MII oocytes that were vitrified at the GV stage, warmed and matured in vitro	Bisulfite mutagenesis and sequencing	*H19* and *KCNQ1OT1*	Oocyte vitrification at the GV stage does not affect the methylation profiles of H19-DMR and KCNQ1OT1-DMR of the in vitro matured oocytes
Zeng et al. [84]	pPig	Spermatozoa	q-PCR and ELISA	*Igf2*	*Igf2* was significantly decreased after vitrification
Zhao et al. [103]	Bovine	Vitrified MII oocytes from matured in vitro	Single-cell whole-genome methylation sequencing	Global analysis	*Peg3* methylation level was significantly decreased in derived blastocysts
Chen et al. [99]	Bovine	Vitrified MII oocytes from matured in vitro	q-PCR	*Peg3, Peg10, Kcnq1ot1, Xist, Igf2r*	*Peg10, Kcnq1ot1, Xist* were significantly increased after vitrification
Chen et al. [100]	Mouse	MII oocytes and 2-cell embryos	q-PCR and bisulfite sequencing	*Gtl2, H19, Igf2, Peg3, Peg10, Igf2r*	*Peg3, Peg10, Igf2r* were significantly different in MII oocytes and 2-cell embryos after vitrification
Cantatore et al. [101]	Mouse	2-cell and blastocyst from vitrified metaphase II oocytes	q-PCR	*Igf2r* and *Gtl2*	No significant differences
Cheng et al. [102]	Mouse	Blastocysts from vitrified MII oocytes	Bisulfite sequencing	*H19, Peg3, Snrpn*	No significant differences in oocytes. Decrease in blastocysts after oocyte vitrification
Ma et al. [98]	Mouse	Mature metaphase II (MII) oocytes	WGBS combined with RNA-seq	Global analysis	*Kcnq1ot1* was significantly down regulated in the vitrified oocytes

DNA methylation is an important modification to regulate imprinted genes. A study have found that DMRs methylation of imprinted genes *H19, Peg3* and *Snrpn* is decreased in blastocysts obtained by in vitro fertilization of vitrified mouse oocytes [102]. Single-cell genome-wide methylation sequencing identified 151 DMRs between the control IVF blastocysts and those obtained after vitrification of bovine MII oocytes, including a DMR located in the maternal imprinted gene *Peg3,* whose methylation level was abnormally reduced by oocyte vitrification [103]. Studies have shown that vitrification of mouse oocytes leads to a decrease in the overall DNA methylation level in oocytes and early embryos [104]. Single-cell whole-genome methylation sequencing (scWGBS) combined with single-cell RNA sequencing (scRNA-seq) also showed that, after the vitrification and warming of mouse MII oocytes, the overall methylation level of the genome decreased abnormally and the expression of the DNA methyltransferase *Dnmt1* decreased significantly [98]. DNMTs expression in vitrified oocytes and the expression of Dnmt3b in blastocysts derived from vitrified oocytes were significantly reduced [102]. Therefore, oocyte vitrification may affect the normal expression of imprinted genes by changing the DNA methylation level of the whole and the regulatory region of imprinted genes.

Different from animal research results, few studies have investigated the effect of human oocyte vitrification on imprinted genes, mainly due to the limited number of human oocytes for research and ethical restrictions on fertilization and embryo processing, which affect the scope and depth of detection. Current studies on vitrification of human oocytes suggest that imprinted genes and DNA methylation are not significantly affected (Table 1). The pyrosequencing method found that the methylation of imprinted genes *H19* and *KCNQ1OT1* was not affected in in vitro maturated MII oocytes derived from vitrified GV oocytes [105]. Immunofluorescence using anti-5-methylcytosine antibody has shown that vitrification and warming of MII oocytes matured in vitro did not affect the overall methylation level of oocytes [106]; further, it found that there was no significant change in the overall DNA methylation level of in vitro cultured 8-cell embryos derived from vitrified oocytes [107]. In a word, the current detection methods have low accuracy and resolution and further research is required.

### Effects of embryo vitrification on imprinted genes

In recent years, many studies have found that the vitrification of embryos has a great impact on imprinted genes in preimplantation embryos, placenta and fetal tissues (Table 2). Porcine blastocyst vitrification leads to a decrease in the expression of imprinted genes *Igf2* and *Igf2*r [108]. In the blastocysts obtained after mouse 2-cell embryo vitrification, the expression of *Gtl2* was downregulated and the expression of *Dlk1, H19* and *Mest* were upregulated [109, 110]. After the mouse 8-cell embryo’s vitrification, the transcription and ICR methylation levels of the imprinted gene *Grb10* were both reduced [111]. *Grb10* is an imprinted gene that regulates social behavior [112]; it can encode growth factor receptor binding protein 10, and its expression is very important for fetal growth. After vitrified mouse embryo transfer, the expression of imprinted genes in the E9.5-day fetus and placenta were detected. It was found that the expression of imprinted genes in fetus (*Sgce, Dcn, Gatm, Gtl2*) and placenta (*Kcnq1ot1, Sgce, Gatm*) had changed, and the methylation level of the *KvDMR1* in fetus and placenta was changed [113]. Another study also detected the effect of mouse embryo vitrification on *H19/Igf2* in the fetus and placenta; they found that the expression of *H19* was abnormally increased and the expression of *Igf2* was abnormally decreased [114]. After the mouse 8-cell embryo’s vitrification, it was found that the overall DNA methylation level decreased abnormally in the warmed 8-cell embryos and the developed blastocysts [111]. After vitrified mouse embryo transfer, the expression of DNA-methylation-related enzymes (*Dnmt1, Dnmt3b, Tet2, Tet3*) was abnormal in the E9.5-day fetus and placenta [113]. Therefore, animal embryo vitrification affects the whole DNA methylation, which may lead to abnormal expression and methylation of some imprinted genes in preimplantation and postimplantation fetus and placenta.

**Table 2:** Effects of embryo cryopreservation on imprinted genes

Reference	Species	Materials	Technology of assessment	Targeted imprinted genes	Conclusions
Derakhshan-Horeh et al. [115]	Human	Blastocysts	Bisulfite sequencing PCR	*H19* and *IGF2*	No significant differences
Yao et al. [116]	Human	Placenta from vitrified embryos	q-PCR, western blot and pyrosequencing	*SNRPN*	The expression level of SNRPN increased after vitrification
Barberet et al. [118]	Human	Placenta	Pyrosequencing and q-PCR	*H19, IGF2, KCNQ1OT1, SNURF*	The placental DNA methylation levels of *H19/IGF2* were lower in the fresh embryo transfer group than in the control (*H19/IGF2*-seq1) and frozen embryo transfer (*H19/IGF2*-seq2) groups
Bartolac et al. [108]	Pig	Blastocysts	q-PCR	*Igf2* and *Igf2r*	*Igf2* and *Igf2r* were significantly decreased after vitrification
Movahed et al. [109]	Mouse	Blastocysts from vitrified 2-cell embryos	q-PCR	*Gtl2* and *Dlk1*	*Gtl2* was down-regulated and *Dlk1* was up-regulated after vitrification
Jahangiri et al. [110]	Mouse	Blastocysts from vitrified 2-cell embryos	q-PCR	*H19* and *Mest*	Significantly increased after vitrification
Yao et al. [111]	Mouse	8-cell embryos	q-PCR and bisulfite sequencing PCR	*Grb10*	The methylation level and gene expression of *Grb10* decreased
Ma et al. [113]	Mouse	Fetuses and placentas from vitrified eight‐cell embryos	q-PCR	*Dlk1, Igf2, Kcnq1ot1, Mest, Ndn, Peg3, Plagl1, Sgce, Snrpn, Cd81, Cdknic, Dcn, Gatm, Gnas, Grb10, Gtl2, H19, Igf2r, Mash2, Osbp15, Phlda2, Slc22a18, Ube3a, Zim1*	A majority of maternally expressed genes were upregulated in fetuses
Wang et al. [114]	Mouse	Fetuses and placentas from vitrified embryos	q-PCR and pyrosequencing	*H19* and *Igf2*	The expression of *H19* was significantly increased after vitrification, whereas *Igf2* was significantly decreased. Methylation levels were decreased

Due to the small number of human embryos and ethical restrictions, it is difficult to obtain enough embryos and tissue materials for research purposes, which hinders study of the impact of human embryo vitrification on imprinted genes. Studies have shown that the vitrification of day-3 human embryos has no significant effect on the DNA methylation status of *H19/IGF2* DMR at the blastocyst stage [115], but the vitrification of human embryos leads to an abnormal increase in the expression of *SNRPN* in placenta [116]. A follow-up study from France showed that, compared with the natural pregnant group, fresh embryo transfer (Fresh-ET) significantly increased the risk of imprint-related diseases (1.6 times higher), but frozen embryo transfer (FET) reduced this risk, which was not statistically different from the natural pregnant group (1.2 times higher) [117]; this may be because the FET cycle can avoid endometrial exposure to high concentrations of gonadotropins, thereby reducing placental dysfunction and pathological changes. However, it is a study based on an epidemiological study. The cases counted as imprinting abnormalities in this study include uniparental disomy, chromosomal structural abnormalities and epimutations. In addition, it is unclear whether the results are based on genetic testing. Uniparental disomy is a chromosomal abnormality, and the usage of controlled ovarian stimulation may increase the frequency of uniparental disomy. A recent study showed that, compared with the natural pregnancy group, the methylation levels of imprinted gene *H19/IGF2* and transposon element *LINE-1* in the placenta of the fresh embryo transfer group were abnormally reduced, but there was no significant abnormality in the FET group [118]. In the future, we need to obtain more preimplantation embryos, placental tissues and aborted fetuses within the scope of ethics without affecting the reproductive needs of patients to clarify the potential adverse effects of embryo vitrification on genomic imprinting through microsample analysis.

### Effects of ovarian tissue cryopreservation on imprinted genes

At present, a few studies on the effects of ovarian tissue cryopreservation on imprinted genes exist (Table 3). After slow freezing and transplantation of mouse ovarian tissue, the ICRs methylation of imprinted genes *H19* and *Kcnq1ot1* in offspring tissue is not affected [119]. After vitrification and warming of ovarian tissues from 10-day-old female mouse, the expression of DNA methyltransferase *Dnmt1* was decreased while imprinted gene *Grb10* was increased [120]. Allogeneic heterotopic transplantation after the vitrification and warming of prepubertal mouse ovarian tissue can restore puberty and fertility, and this process does not affect the DMR methylation of imprinted gene *Snrpn* in GV oocytes [121]. After vitrification and warming of mouse follicles, the DMR methylation of imprinted genes *H19* and *Igf2r* in mature oocytes was not affected, and *Snrpn* changed slightly [122]. Vitrification and warming of mouse ovarian tissue affected follicular growth, and the promoter methylation level of *Inhba* in granulosa cells decreased [123]. In summary, there are limited studies investigating the influences of ovarian tissue vitrification on imprinted genes in mouse and there is still a lack of research on human ovarian tissue.

**Table 3:** Effects of ovarian tissue cryopreservation on imprinted genes

Reference	Species	Materials	Technology of assessment	Targeted imprinted genes	Conclusions
Sauvat et al. [119]	mouse	ovarian tissue	Southern blotting	*H19* and *Kcnq1ot1*	No significant differences
He et al. [120]	mouse	ovarian tissue	Western blot and q-PCR	*Grb10*	*Grb10* was significantly increased after vitrification
Wang et al. [121]	mouse	ovarian tissue	Bisulfite sequencing PCR	*Snrpn*	Freezing of ovarian tissue did not affect the methylation status of *Snrpn*-DMR of imprinted genes in GV oocytes
Trapphoff et al. [122]	mouse	pre-antral follicles	pyrosequencing	*H19, Igf2* and *Snrpn*	The methylation of DMR of imprinted gene *H19* and *Igf2r* in mature oocytes was not affected, and *Snrpn* changed slightly

## Conclusion

At present, oocyte and embryo vitrification is widely used in ART. Although ovarian tissue cryopreservation is developed late and slowly, the vitrification method has developed rapidly. Currently, strategies for sperm cryopreservation are still mainly slow or rapid freezing, and the vitrification technique requires further development and evaluation. Compared with traditional freezing methods, the vitrification process is time-consuming and convenient. Most importantly, it can minimize the damage to the ultrastructure and nuclear DNA in gametes, embryos and follicles and improve the survival and clinical outcomes.

Vitrification can significantly affect the expression and methylation of some imprinted genes in animal oocytes and embryos, and the few studies in humans have reported almost no influence. The slow or rapid freezing of human sperm does not affect the DNA methylation related to imprinted genes, but the effect of vitrification is unknown. There is still a lack of research on the effect of vitrification on imprinted genes in human ovarian tissue. In summary, vitrification can adversely affect imprinted genes in animals, but the impacts of vitrification on human imprinted genes have not been fully studied. It is necessary to strengthen the detection of the effects of vitrification on imprinted genes in human gametes, embryos and tissues and to conduct a long-term follow-up study to evaluate the safety for their offspring.

## Data Availability

Not applicable.

## Abbreviations

ART: Assisted reproductive technology
BWS: Beckwith–Wiedemann
PWS: Prader–Willi syndrome
AS: Angelman syndrome
PGCs: Primordial germ cells
ICRs: Imprinting control regions
DMRs: Differentially methylated regions
OTC: Ovarian tissue cryopreservation
scWGBS: Single-cell whole-genome methylation sequencing
scRNA-seq: Single-cell RNA SEQ
FET: Frozen embryo transfer
5mC: 5-Methylcytosine

## References

[1] Fesahat F, Montazeri F, Hoseini SM (2020). Preimplantation genetic testing in assisted reproduction technology. J Gynecol Obstet Hum Reprod.

[2] Esteves SC, Humaidan P, Roque M, Agarwal A (2019). Female infertility and assisted reproductive technology. Panminerva Med.

[3] Agarwal A, Esteves SC, Humaidan P, Roque M (2019). Male infertility and assisted reproductive technology. Panminerva Med.

[4] Mukaida T, Wada S, Takahashi K, Pedro P, An T, Kasai M (1998). Vitrification of human embryos based on the assessment of suitable conditions for 8-cell mouse embryos. Hum Reprod.

[5] Kuleshova L, Gianaroli L, Magli C, Ferraretti A, Trounson A (1999). Birth following vitrification of a small number of human oocytes: case report. Hum Reprod.

[6] Kometas M, Christman GM, Kramer J, Rhoton-Vlasak A (2021). Methods of ovarian tissue cryopreservation: is vitrification superior to slow freezing?—Ovarian tissue freezing methods. Reprod Sci.

[7] Ramos L, Galbinski S, Nacul A, Jiménez MF, Frantz N, Bos-Mikich A, Detailed morphological analysis of cryoinjury in human ovarian tissue following vitrification or slow freezing. 2021. 10.1007/s43032-021-00716-x 34398410

[8] Arshad U, Sagheer M, González-Silvestry FB, Hassan M, Sosa F (2021). Vitrification improves in-vitro embryonic survival in Bos taurus embryos without increasing pregnancy rate post embryo transfer when compared to slow-freezing: a systematic meta-analysis. Cryobiology.

[9] Ahuja KK, Macklon N (2020). Vitrification and the demise of fresh treatment cycles in ART. Reprod Biomed Online.

[10] Cornet-Bartolomé D, Rodriguez A, García D, Barragán M, Vassena R (2020). Efficiency and efficacy of vitrification in 35,654 sibling oocytes from donation cycles. Hum Reprod.

[11] Cao Y, Xing Q, Zhang ZG, Wei ZL, Zhou P, Cong L (2009). Cryopreservation of immature and in-vitro matured human oocytes by vitrification. Reprod Biomed Online.

[12] Hezavehei M, Sharafi M, Kouchesfahani HM, Henkel R, Agarwal A, Esmaeili V, Shahverdi A (2018). Sperm cryopreservation: a review on current molecular cryobiology and advanced approaches. Reprod Biomed Online.

[13] Barberet J, Barry F, Choux C, Guilleman M, Karoui S, Simonot R, Bruno C, Fauque P (2020). What impact does oocyte vitrification have on epigenetics and gene expression?. Clin Epigenet.

[14] Yodrug T, Parnpai R, Hirao Y, Somfai T (2021). Effect of vitrification at different meiotic stages on epigenetic characteristics of bovine oocytes and subsequently developing embryos. Anim Sci J.

[15] Moulavi F, Saadeldin I, Swelum A, Tasdighi F, Hosseini-Fahraji H, Hosseini S (2021). Oocyte vitrification induces loss of DNA methylation and histone acetylation in the resulting embryos derived using ICSI in dromedary camel. Zygote.

[16] Renfree MB, Hore TA, Shaw G, Graves JA, Pask AJ (2009). Evolution of genomic imprinting: insights from marsupials and monotremes. Annu Rev Genomics Hum Genet.

[17] Anvar Z, Chakchouk I, Demond H (2021). DNA methylation dynamics in the female germline and maternal-effect mutations that disrupt genomic imprinting. Zygote.

[18] Kaneko-Ishino T, Ishino F (2022). The evolutionary advantage in mammals of the complementary monoallelic expression mechanism of genomic imprinting and its emergence from a defense against the insertion into the host genome. Front Genet.

[19] Isles AR (2022). The contribution of imprinted genes to neurodevelopmental and neuropsychiatric disorders. Transl Psychiatry.

[20] Sandovici I, Georgopoulou A, Pérez-García V, Hufnagel A, López-Tello J, Lam BYH, Schiefer SN, Gaudreau C, Santos F, Hoelle K, Yeo GSH, Burling K, Reiterer M, Fowden AL, Burton GJ, Branco CM, Sferruzzi-Perri AN, Constância M (2022). The imprinted Igf2-Igf2r axis is critical for matching placental microvasculature expansion to fetal growth. Dev Cell.

[21] Smith FM, Garfield AS, Ward A (2006). Regulation of growth and metabolism by imprinted genes. Cytogenet Genome Res.

[22] Millership SJ, Van de Pette M, Withers DJ (2019). Genomic imprinting and its effects on postnatal growth and adult metabolism. Zygote.

[23] Mangiavacchi PM, Caldas-Bussiere MC, Mendonça MDS, Dias AJB, Rios ÁFL (2021). Multi-locus imprinting disturbances of Beckwith–Wiedemann and large offspring syndrome/abnormal offspring syndrome: a brief review. Theriogenology.

[24] Lim DH, Maher ER (2010). Genomic imprinting syndromes and cancer. Adv Genet.

[25] Elhamamsy AR (2017). Role of DNA methylation in imprinting disorders: an updated review. J Assist Reprod Genet.

[26] Hanna CW, Kelsey G (2021). Features and mechanisms of canonical and noncanonical genomic imprinting. Adv Genet.

[27] Kelsey G, Feil R, New insights into establishment and maintenance of DNA methylation imprints in mammals. Philos Trans R Soc Lond Ser B Biol Sci. 2013:368(1609):20110336. 10.1098/rstb.2011.0336 PMC3539362 23166397

[28] Monk D (2015). Germline-derived DNA methylation and early embryo epigenetic reprogramming: the selected survival of imprints. Int J Biochem Cell Biol.

[29] SanMiguel JM, Bartolomei MS (2018). DNA methylation dynamics of genomic imprinting in mouse development. Biol Reprod.

[30] Ivanova E, Canovas S, Garcia-Martínez S, Romar R, Lopes JS, Rizos D, Sanchez-Calabuig MJ, Krueger F, Andrews S, Perez-Sanz F, Kelsey G, Coy P (2020). DNA methylation changes during preimplantation development reveal inter-species differences and reprogramming events at imprinted genes. Biol Reprod.

[31] Szabó PE, Hübner K, Schöler H, Mann JR (2002). Allele-specific expression of imprinted genes in mouse migratory primordial germ cells. Mech Dev.

[32] Lee J, Inoue K, Ono R, Ogonuki N, Kohda T, Kaneko-Ishino T, Ogura A, Ishino F, Erasing genomic imprinting memory in mouse clone embryos produced from day 11.5 primordial germ cells. 2002. 10.1242/dev.129.8.1807 11934847

[33] Stewart KR, Veselovska L, Kelsey G (2016). Establishment and functions of DNA methylation in the germline. Epigenomics.

[34] Hanna CW, Demond H, Kelsey G (2018). Epigenetic regulation in development: is the mouse a good model for the human?. Hum Reprod Update.

[35] Monk D, Mackay DJG, Eggermann T, Maher ER, Riccio A (2019). Genomic imprinting disorders: lessons on how genome, epigenome and environment interact. Nat Rev Genet.

[36] Barberet J, Binquet C, Guilleman M, Doukani A, Choux C, Bruno C, Bourredjem A, Chapusot C, Bourc'his D, Duffourd Y, Fauque P (2021). Do assisted reproductive technologies and in vitro embryo culture influence the epigenetic control of imprinted genes and transposable elements in children?. Hum Reprod.

[37] Ochoa E (2021). Alteration of genomic imprinting after assisted reproductive technologies and long-term health. Life.

[38] Denomme MM, Mann MR (2012). Genomic imprints as a model for the analysis of epigenetic stability during assisted reproductive technologies. Reproduction.

[39] Sciorio R, El Hajj N (2022). Epigenetic risks of medically assisted reproduction. J Clin Med.

[40] DeBaun MR, Niemitz EL, Feinberg AP (2003). Association of in vitro fertilization with Beckwith–Wiedemann syndrome and epigenetic alterations of LIT1 and H19. Am J Hum Genet.

[41] Gicquel C, Gaston V, Mandelbaum J, Siffroi J-P, Flahault A, Le Bouc Y, In vitro fertilization may increase the risk of Beckwith–Wiedemann syndrome related to the abnormal imprinting of the KCNQ1OT gene. Am J Hum Genet. 2003;72(5):1338. 10.1086/374824 PMC1180288 12772698

[42] Halliday J, Oke K, Breheny S, Algar E, Amor DJ (2004). Beckwith–Wiedemann syndrome and IVF: a case-control study. Am J Hum Genet.

[43] Gomes MV, Huber J, Ferriani RA, Amaral Neto AM, Ramos ES (2009). Abnormal methylation at the KvDMR1 imprinting control region in clinically normal children conceived by assisted reproductive technologies. Mol Hum Reprod.

[44] Mani S, Ghosh J, Coutifaris C, Sapienza C, Mainigi M (2020). Epigenetic changes and assisted reproductive technologies. Epigenetics.

[45] DeAngelis AM, Martini AE, Owen CM (2018). Assisted Reproductive Technology and Epigenetics. Seminars in reproductive medicine.

[46] Rall WF, Fahy GM (1985). Ice-free cryopreservation of mouse embryos at −196 °C by vitrification. Nature.

[47] Nakagata N (1989). High survival rate of unfertilized mouse oocytes after vitrification. J Reprod Fertil.

[48] El Cury-Silva T, Nunes ME, Casalechi M, Comim FV, Rodrigues JK, Reis FM (2021). Cryoprotectant agents for ovarian tissue vitrification: systematic review. Cryobiology.

[49] Li J, Xiong S, Zhao Y, Li C, Han W, Huang G (2021). Effect of the re-vitrification of embryos at different stages on embryonic developmental potential. Front Endocrinol.

[50] Cobo A, García-Velasco JA, Remohí J, Pellicer A (2021). Oocyte vitrification for fertility preservation for both medical and nonmedical reasons. Fertil Steril.

[51] Koohestanidehaghi Y, Torkamanpari M, Shirmohamadi Z, Lorian K, Vatankhah M (2021). The effect of cysteine and glutamine on human sperm functional parameters during vitrification. Andrologia.

[52] Nur Karakus F, Bulgurcuoglu Kuran S, Solakoglu S (2021). Effect of curcumin on sperm parameters after the cryopreservation. Eur J Obstet Gynecol Reprod Biol.

[53] Isachenko E, Isachenko V, Weiss JM, Kreienberg R, Katkov II, Schulz M, Lulat AG, Risopatron MJ, Sanchez R (2008). Acrosomal status and mitochondrial activity of human spermatozoa vitrified with sucrose. Reproduction.

[54] Riva NS, Ruhlmann C, Iaizzo RS, Marcial Lopez CA, Martinez AG (2018). Comparative analysis between slow freezing and ultra-rapid freezing for human sperm cryopreservation. JBRA assisted reproduction.

[55] Aizpurua J, Medrano L, Enciso M, Sarasa J, Romero A, Fernandez MA, Gomez-Torres MJ (2017). New permeable cryoprotectant-free vitrification method for native human sperm. Hum Reprod.

[56] Sharma R, Kattoor AJ, Ghulmiyyah J, Agarwal A (2015). Effect of sperm storage and selection techniques on sperm parameters. Syst Biol Reprod Med.

[57] Hu H, Ji G, Shi X, Liu R, Zhang J, Zhang H, Yuan X, Zhang G, Yuan W, Li M (2020). Comparison of rapid freezing versus vitrification for human sperm cryopreservation using sucrose in closed straw systems. Cell Tissue Bank.

[58] Amer M, Ismail N, Gamal El Din SF, Rashad EZ, Fakhry E, Abd El Hakim W, Ragab A (2019). 2019 Effect of cryoprotectant-free vitrification versus conventional freezing on human testicular sperm motility: a prospective comparative study. Hum Fertil.

[59] Li Y-X, Zhou L, Lv M-Q, Ge P, Liu Y-C, Zhou D-X (2019). Vitrification and conventional freezing methods in sperm cryopreservation: a systematic review and meta-analysis. Eur J Obstet Gynecol Reprod Biol.

[60] Rienzi L, Gracia C, Maggiulli R, LaBarbera AR, Kaser DJ, Ubaldi FM, Vanderpoel S, Racowsky C (2017). Oocyte, embryo and blastocyst cryopreservation in ART: systematic review and meta-analysis comparing slow-freezing versus vitrification to produce evidence for the development of global guidance. Hum Reprod Update.

[61] Debrock S, Peeraer K, Fernandez Gallardo E, De Neubourg D, Spiessens C, D'Hooghe TM (2015). Vitrification of cleavage stage day 3 embryos results in higher live birth rates than conventional slow freezing: a RCT. Hum Reprod.

[62] Mature oocyte cryopreservation (2013). a guideline. Fertil Steril.

[63] Cobo A, Serra V, Garrido N, Olmo I, Pellicer A, Remohi J, Obstetric and perinatal outcome of babies born from vitrified oocytes. Fertil Steril. 2014;102(4):1006–1015e4. 10.1016/j.fertnstert.2014.06.019 25064408

[64] Martinez M, Rabadan S, Domingo J, Cobo A, Pellicer A, Garcia-Velasco JA (2014). Obstetric outcome after oocyte vitrification and warming for fertility preservation in women with cancer. Reprod Biomed Online.

[65] Henry L, Labied S, Jouan C, Nisolle M (2022). Preservation of female fertility: The current therapeutic strategy. Int J Gynaecol Obstet.

[66] Maggiulli R, Vaiarelli A, Cimadomo D, Giancani A, Tacconi L, Fabozzi G, Ubaldi FM, Rienzi L (2021). Fertility preservation through oocyte vitrification: clinical and laboratory perspectives. J Vis Exp.

[67] Dolmans MM, Manavella DD (2019). Recent advances in fertility preservation. J Obstet Gynaecol Res.

[68] Nagy ZP, Shapiro D, Chang CC (2020). Vitrification of the human embryo: a more efficient and safer in vitro fertilization treatment. Fertil Steril.

[69] Sung H, Ferlay J, Siegel RL, Laversanne M, Soerjomataram I, Jemal A, Bray F (2021). Global cancer statistics 2020: GLOBOCAN estimates of incidence and mortality worldwide for 36 cancers in 185 countries. Cancer J Clin.

[70] Bray F, Ferlay J, Soerjomataram I, Siegel RL, Torre LA, Jemal A (2018). Global cancer statistics 2018: GLOBOCAN estimates of incidence and mortality worldwide for 36 cancers in 185 countries. Cancer J Clin.

[71] Sonigo C, Beau I, Binart N, Grynberg M (2019). The impact of chemotherapy on the ovaries: molecular aspects and the prevention of ovarian damage. Int J Mol Sci.

[72] Gargus E, Deans R, Anazodo A, Woodruff TK (2018). Management of primary ovarian insufficiency symptoms in survivors of childhood and adolescent cancer. J Natl Compr Canc Netw.

[73] Donnez J, Dolmans M-M (2017). Fertility preservation in women. N Engl J Med.

[74] Medicine PC (2019). Fertility preservation in patients undergoing gonadotoxic therapy or gonadectomy: a committee opinion. Fertil Steril.

[75] Dolmans MM, von Wolff M, Poirot C, Diaz-Garcia C, Cacciottola L, Boissel N, Liebenthron J, Pellicer A, Donnez J, Andersen CY (2021). Transplantation of cryopreserved ovarian tissue in a series of 285 women: a review of five leading European centers. Fertil Steril.

[76] Shi Q, Xie Y, Wang Y, Li S (2017). Vitrification versus slow freezing for human ovarian tissue cryopreservation: a systematic review and meta-anlaysis. Scientific reports.

[77] El Cury-Silva T, Nunes MEG, Casalechi M, Comim FV, Rodrigues JK, Reis FM (2021). Cryoprotectant agents for ovarian tissue vitrification: Systematic review. Cryobiology.

[78] Rivas Leonel EC, Lucci CM, Amorim CA (2019). Cryopreservation of human ovarian tissue: a review. Transfusion medicine and hemotherapy.

[79] Kawamura K, Cheng Y, Suzuki N, Deguchi M, Sato Y, Takae S, Ho CH, Kawamura N, Tamura M, Hashimoto S, Sugishita Y, Morimoto Y, Hosoi Y, Yoshioka N, Ishizuka B, Hsueh AJ (2013). Hippo signaling disruption and Akt stimulation of ovarian follicles for infertility treatment. Proc Natl Acad Sci USA.

[80] Suzuki N, Yoshioka N, Takae S, Sugishita Y, Tamura M, Hashimoto S, Morimoto Y, Kawamura K (2015). Successful fertility preservation following ovarian tissue vitrification in patients with primary ovarian insufficiency. Hum Reprod.

[81] Santi D, De Vincentis S, Magnani E, Spaggiari G (2017). Impairment of sperm DNA methylation in male infertility: a meta-analytic study. Andrology.

[82] Peng H, Zhao P, Liu J, Zhang J, Zhang J, Wang Y, Wu L, Song M, Wang W (2018). Novel epigenomic biomarkers of male infertility identified by methylation patterns of CpG sites within imprinting control regions of H19 and SNRPN genes. OMICS.

[83] Costes V, Chaulot-Talmon A, Sellem E (2022). Predicting male fertility from the sperm methylome: application to 120 bulls with hundreds of artificial insemination records. Cryobiology.

[84] Zeng C, Peng W, Ding L, He L, Zhang Y, Fang D, Tang K (2014). A preliminary study on epigenetic changes during boar spermatozoa cryopreservation. Cryobiology.

[85] Flores E, Ramió-Lluch L, Bucci D, Fernández-Novell J, Peña A, Rodríguez-Gil J (2011). Freezing-thawing induces alterations in histone H1-DNA binding and the breaking of protein-DNA disulfide bonds in boar sperm. Theriogenology.

[86] Jia G, Fu X, Cheng K, Yue M, Jia B, Hou Y, Zhu S (2015). Spermatozoa cryopreservation alters pronuclear formation and zygotic DNA demethylation in mice. Theriogenology.

[87] Chao S, Li J, Jin X, Tang H, Wang G, Gao G (2012). Epigenetic reprogramming of embryos derived from sperm frozen at − 20°C. Sci China Life Sci.

[88] de Oliveira RA, Scarlet D, Ille N, Aurich C (2017). Cooled-storage of equine semen does not induce major changes in sperm DNA methylation. Theriogenology.

[89] Aurich C, Schreiner B, Ille N, Alvarenga M, Scarlet D (2016). Cytosine methylation of sperm DNA in horse semen after cryopreservation. Theriogenology.

[90] Kläver R, Bleiziffer A, Redmann K, Mallidis C, Kliesch S, Gromoll J (2012). Routine cryopreservation of spermatozoa is safe—evidence from the DNA methylation pattern of nine spermatozoa genes. J Assist Reprod Genet.

[91] Khosravizadeh Z, Hassanzadeh G, Tavakkoly Bazzaz J, Alizadeh F, Totonchi M, Salehi E, Khodamoradi K, Khanehzad M, Hosseini SR, Abolhassani F (2020). The effect of cryopreservation on DNA methylation patterns of the chromosome 15q11-q13 region in human spermatozoa. Cell Tissue Bank.

[92] Park JK, Lee JH (2021). Development of optimized vitrification procedures using closed carrier system to improve the survival and developmental competence of vitrified mouse oocytes. Cell.

[93] Viana IGR, Vireque AA, Navarro PA (2022). Comparing the effects of a commercial and a prototype vitrification medium on meiotic spindle morphology and survival rate of mouse oocytes. Cell.

[94] Girka E, Gatenby L, Gutierrez EJ, Bondioli KR (2022). The effects of microtubule stabilizing and recovery agents on vitrified bovine oocytes. Theriogenology.

[95] Khalili MA, Maione M, Palmerini MG, Bianchi S, Macchiarelli G, Nottola SA (2022). Ultrastructure of human mature oocytes after vitrification. Eur J histochem.

[96] Palmerini MG, Antinori M, Maione M, Cerusico F, Versaci C, Nottola SA, Macchiarelli G, Khalili MA, Antinori S (2014). Ultrastructure of immature and mature human oocytes after cryotop vitrification. J Reprod Dev.

[97] Hwang I-S, Kwon D-J, Im G-S, Tashima K, Hochi S, Hwang S (2016). High incidence of polyspermic fertilization in bovine oocytes matured in vitro after Cryotop vitrification. CryoLetters.

[98] Ma Y, Long C, Liu G, Bai H, Ma L, Bai T, Zuo Y, Li S (2021). WGBS combined with RNA-seq analysis revealed that Dnmt1 affects the methylation modification and gene expression changes during mouse oocyte vitrification. Theriogenology.

[99] Chen H, Zhang L, Deng T, Zou P, Wang Y, Quan F, Zhang Y (2016). Effects of oocyte vitrification on epigenetic status in early bovine embryos. Theriogenology.

[100] Chen H, Zhang L, Wang Z, Chang H, Xie X, Fu L, Zhang Y, Quan F (2019). Resveratrol improved the developmental potential of oocytes after vitrification by modifying the epigenetics. Mol Reprod Dev.

[101] Cantatore C, George JS, Depalo R, D’Amato G, Moravek M, Smith GD (2021). Mouse oocyte vitrification with and without dimethyl sulfoxide: influence on cryo-survival, development, and maternal imprinted gene expression. J Assist Reprod Genet.

[102] Cheng KR, Fu XW, Zhang RN, Jia GX, Hou YP, Zhu SE (2014). Effect of oocyte vitrification on deoxyribonucleic acid methylation of H19, Peg3, and Snrpn differentially methylated regions inmouse blastocysts. Fertil Steril.

[103] Zhao YH, Wang JJ, Zhang PP, Hao HS, Pang YW, Wang HY, Du WH, Zhao SJ, Ruan WM, Zou HY, Hao T, Zhu HB, Zhao XM (2020). Oocyte IVM or vitrification significantly impairs DNA methylation patterns in blastocysts as analysed by single-cell whole-genome methylation sequencing. Reprod Fertil Dev.

[104] Ying L, Xiang-Wei F, Jun-Jie L, Dian-Shuai Y, Shi-En Z (2014). DNA methylation pattern in mouse oocytes and their in vitro fertilized early embryos: effect of oocyte vitrification. Zygote.

[105] Al-Khtib M, Perret A, Khoueiry R, Ibala-Romdhane S, Blachère T, Greze C, Lornage J, Lefèvre A (2011). Vitrification at the germinal vesicle stage does not affect the methylation profile of H19 and KCNQ1OT1 imprinting centers in human oocytes subsequently matured in vitro. Fertil Steril.

[106] Liu M-H, Zhou W-H, Chu D-P, Fu L, Sha W, Li Y (2017). Ultrastructural changes and methylation of human oocytes vitrified at the germinal vesicle stage and matured in vitro after thawing. Gynecol Obstet Inves.

[107] DeMunck N, Petrussa L, Verheyen G, Staessen C, Vandeskelde Y, Sterckx J, Bocken G, Jacobs K, Stoop D, De Rycke M (2015). Chromosomal meiotic segregation, embryonic developmental kinetics and DNA (hydroxy) methylation analysis consolidate the safety of human oocyte vitrification. Basic Sci Reprod Med.

[108] Bartolac LK, Lowe JL, Koustas G, Grupen CG, Sjöblom C (2018). Vitrification, not cryoprotectant exposure, alters the expression of developmentally important genes in in vitro produced porcine blastocysts. Cryobiology.

[109] Movahed E, Shabani R, Hosseini S, Shahidi S, Salehi M (2020). Interfering effects of in vitro fertilization and vitrification on expression of Gtl2 and Dlk1 in mouse blastocysts. Int J Fertil Steril.

[110] Jahangiri M, Shahhoseini M, Movaghar B (2014). H19 and MEST gene expression and histone modification in blastocysts cultured from vitrified and fresh two-cell mouse embryos. Reprod Biomed Online.

[111] Yao J, Geng L, Huang R, Peng W, Chen X, Jiang X, Yu M, Li M, Huang Y, Yang X (2017). Effect of vitrification on in vitro development and imprinted gene Grb10 in mouse embryos. Reproduction.

[112] Garfield AS, Michael C, Smith FM, Kim M, Stewart-Cox JE, Kerry G, Sian B, Jing X, Dalley JW, Hurst LD (2011). Distinct physiological and behavioural functions for parental alleles of imprinted Grb10. Nature.

[113] Ma Y, Ma Y, Wen L, Lei H, Chen S, Wang X (2019). Changes in DNA methylation and imprinting disorders in E9. 5 mouse fetuses and placentas derived from vitrified 8-cell embryos. Mol Reprod. Dev.

[114] Wang Z, Xu L, He F (2010). Embryo vitrification affects the methylation of the H19/Igf2 differentially methylated domain and the expression of H19 and Igf2. Fertil Steril.

[115] Derakhshan-Horeh M, Abolhassani F, Jafarpour F, Moini A, Karbalaie K, Hosseini SM, Nasr-Esfahani MH (2016). Vitrification at Day3 stage appears not to affect the methylation status of H19/IGF2 differentially methylated region of in vitro produced human blastocysts. Cryobiology.

[116] Yao J-F, Huang Y-F, Huang R-F, Lin S-X, Guo C-Q, Hua C-Z, Wu P-Y, Hu J-F, Li Y-Z (2020). Effects of vitrification on the imprinted gene Snrpn in neonatal placental tissue. Reprod Dev Med.

[117] Fauque P, De Mouzon J, Devaux A, Epelboin S, Gervoise-Boyer MJ, Levy R, Valentin M, Viot G, Bergere A, DeVienne C, Jonveaux P, Pessione F (2020). Reproductive technologies, female infertility, and the risk of imprinting-related disorders. Clin Epigenet.

[118] Barberet J, Romain G, Binquet C, Guilleman M, Bruno C, Ginod P, Chapusot C, Choux C, Fauque P (2021). Do frozen embryo transfers modify the epigenetic control of imprinted genes and transposable elements in newborns compared with fresh embryo transfers and natural conceptions?. Fertil Steril.

[119] Sauvat F, Capito C, Sarnacki S, Poirot C, Bachelot A, Meduri G, Dandolo L, Binart N (2008). Immature cryopreserved ovary restores puberty and fertility in mice without alteration of epigenetic marks. PLoS ONE.

[120] He Z-Y, Wang H-Y, Zhou X, Liang X-Y, Yan B, Wang R, Ma L-H, Wang Y-L (2018). Evaluation of vitrification protocol of mouse ovarian tissue by effect of DNA methyltransferase-1 and paternal imprinted growth factor receptor-binding protein 10 on signaling pathways. Cryobiology.

[121] Wang HY, Li YH, Sun L, Gao X, You L, Wang Y, Ma JL, Chen ZJ (2013). Allotransplantation of cryopreserved prepubertal mouse ovaries restored puberty and fertility without affecting methylation profile of Snrpn-DMR. Fertil Steril.

[122] Trapphoff T, El Hajj N, Zechner U, Haaf T, Eichenlaub-Ritter U (2010). DNA integrity, growth pattern, spindle formation, chromosomal constitution and imprinting patterns of mouse oocytes from vitrified pre-antral follicles. Hum Reprod.

[123] Damavandi M, Farrokh P, Zavareh S (2021). Effect of mouse ovarian vitrification on promoter methylation of inhba and inhbb in granulosa cells of follicles. Cryo Lett.

